# Cytosolic Replication of Group A *Streptococcus* in Human Macrophages

**DOI:** 10.1128/mBio.00020-16

**Published:** 2016-04-12

**Authors:** Alan M. O’Neill, Teresa L. M. Thurston, David W. Holden

**Affiliations:** MRC Centre for Molecular Bacteriology and Infection, Imperial College London, United Kingdom

## Abstract

As key components of innate immune defense, macrophages are essential in controlling bacterial pathogens, including group A *Streptococcus* (GAS). Despite this, only a limited number of studies have analyzed the recovery of GAS from within human neutrophils and macrophages. Here, we determined the intracellular fate of GAS in human macrophages by using several quantitative approaches. In both U937 and primary human macrophages, the appearance over time of long GAS chains revealed that despite GAS-mediated cytotoxicity, replication occurred in viable, propidium iodide-negative macrophages. Whereas the major virulence factor M1 did not contribute to bacterial growth, a GAS mutant strain deficient in streptolysin O (SLO) was impaired for intracellular replication. SLO promoted bacterial escape from the GAS-containing vacuole (GCV) into the macrophage cytosol. Up to half of the cytosolic GAS colocalized with ubiquitin and p62, suggesting that the bacteria were targeted by the autophagy machinery. Despite this, live imaging of U937 macrophages revealed proficient replication of GAS after GCV rupture, indicating that escape from the GCV is important for growth of GAS in macrophages. Our results reveal that GAS can replicate within viable human macrophages, with SLO promoting GCV escape and cytosolic growth, despite the recruitment of autophagy receptors to bacteria.

## IMPORTANCE

Classically regarded as an extracellular pathogen, GAS can persist within human epithelial cells, as well as neutrophils and macrophages. Some studies suggest that GAS can modulate its intracellular vacuole to promote survival and perhaps replicate in macrophages. However, an in-depth single-cell analysis of the dynamics of survival and replication is lacking. We used macrophage-like cell lines and primary macrophages to measure the intracellular growth of GAS at both the population and single-cell levels. While CFU counts revealed no increase in overall bacterial growth, quantitative fluorescence microscopy, flow cytometry, and time-lapse imaging revealed bacterial replication in a proportion of infected macrophages. This study emphasizes the importance of single-cell analysis especially when studying the intracellular fate of a pathogen that is cytotoxic and displays heterogeneity in terms of intracellular killing and growth. To our knowledge, this study provides the first direct visualization of GAS replication inside human cells.

## INTRODUCTION

Group A *Streptococcus* (GAS) causes a wide variety of diseases in immunocompetent individuals, from localized skin infections and recurrent bouts of tonsillitis to more life-threatening invasive diseases, such as streptococcal toxic shock syndrome (STSS) and necrotizing fasciitis (NF). Skin and pharyngeal epithelia represent the primary sites of GAS interaction with the host (1). Upon the invasion of epithelial cells, GAS can persist for several days (2, 3). However, many strains fail to proliferate and there is evidence that autophagy and the endolysosomal pathway contribute to the intracellular clearance of GAS (4, 5). Other studies have suggested that internalization of GAS in host cells provides a safe haven from host cell killing and antibiotic-mediated killing *in vivo* (6, 7).

B and T cell-deficient mice exhibit a similar resistance to GAS in comparison to immunocompetent mice (8), but depletion of macrophages causes a substantial increase in bacterial dissemination and mortality (9, 10). However, the contribution of macrophages to disease outcome in humans is not clear. Thulin and colleagues found that macrophages were a primary reservoir of viable GAS in biopsy specimens from patients with invasive disease (11). The presence of a heavy bacterial load despite intravenous antibiotic therapy, suggests that GAS can resist and/or evade macrophage killing mechanisms. This finding was also supported by *in vitro* observations, which implicated the surface protein M1 in avoidance fusion between GAS and azurophilic granules and lysosomes in human neutrophils and monocyte-derived macrophages, respectively (12, 13).

Another important virulence factor in GAS-host interactions is the pore-forming toxin streptolysin O (SLO). SLO is produced and secreted as a monomer that binds cholesterol in cell membranes and oligomerizes to form large transmembrane pores (14). Upon macrophage phagocytosis, SLO activity causes a dose-dependent form of apoptotic cell death among a population of infected cells (15, 16). *In vitro* and *in vivo* data support the hypothesis that SLO-induced toxicity contributes to GAS immune evasion and increased virulence. Moreover, it is becoming increasingly clear that SLO has other important intracellular functions beyond cytotoxicity. SLO has been reported to prevent bacterial internalization in pharyngeal keratinocytes (17) and promote escape from vacuoles in HeLa cells (18). However, it has also been reported that in macrophages, GAS survives within a modified vacuolated compartment that could serve as a replicative niche (13). A more recent study indicated that SLO-mediated pore formation does not promote bacterial escape from the GAS-containing vacuole (GCV) in THP-1 macrophages but rather prevents vacuole acidification (19). However, GAS proliferation in human macrophages has yet to be observed or quantified by single-cell analysis. In this study, we used macrophage-like cell lines and monocyte-derived primary macrophages together with CFU-based assays, quantitative fluorescence microscopy, flow cytometry, and time-lapse imaging to observe and measure the intracellular growth of GAS at both the population and single-cell levels. While CFU counts revealed no increase in overall bacterial growth in these cells, microscopic examination revealed bacterial replication in a proportion of infected primary and U937 macrophages. Replication occurred in the host cell cytosol, after SLO-dependent phagosomal rupture, despite recruitment of autophagy proteins to a subpopulation of cytosolic bacteria. Our results suggest that despite the ability of macrophages to control the net intracellular growth of GAS, cytosolic growth within macrophage subpopulations might contribute to the virulence of this pathogen.

## RESULTS

### M1T1 GAS replicates in U937 and primary human macrophages.

**FIG 1:**
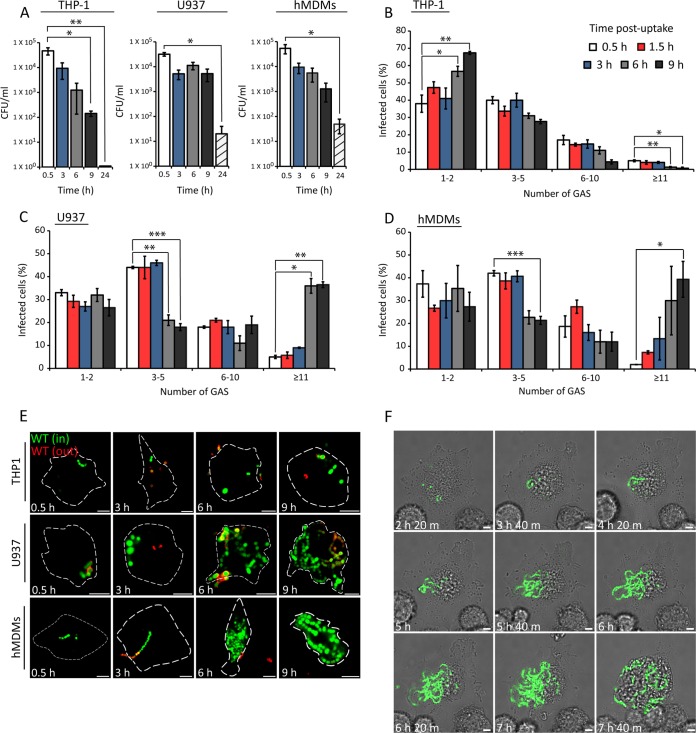
Quantification of intracellular M1T1 GAS in human macrophages. (A) Survival of M1T1 5448 GAS in the cell lines indicated. Cells were infected at an MOI of 5, and intracellular bacteria were measured by CFU counting at the time points indicated. The mean ± SEM of three independent experiments in triplicate wells and duplicate colony counts is shown. (B to D) Quantification of intracellular GFP-M1T1 5448 in the cell lines indicated. Cells were infected at an MOI of 0.5, and the percentage of cells containing 1 or 2, 3 to 5, 6 to 10, or ≥11 bacteria per infected cell was scored by fluorescence microscopy. The numbers of bacteria in at least 100 infected cells per experiment were determined. The mean ± SEM of at least three independent experiments is shown. *, *P* < 0.05; **, *P* < 0.01; ***, *P* < 0.001 (two-tailed paired t test). (E) Representative confocal microscopy images of macrophages infected with GFP-M1T1 5448 GAS at the time points indicated. Intracellular bacteria (in) were discriminated from extracellular bacteria (out) by differential labeling with an anti-GAS primary antibody and an Alexa Fluor 555-conjugated secondary antibody (red) without cell permeabilization (scale bars, 5 µm). Cell outlines are delineated by broken white lines. (F) Stills from live confocal imaging of U937 cells infected with GFP-M1T1 5448 at an MOI of 0.5. Imaging was initiated at 2 hpu, and the cells were imaged every 20 min until 10 hpu. The elapsed time is shown at the bottom left of each image (scale bars, 5 µm).

To investigate if GAS can survive and replicate in human macrophages, THP-1, U937, and human monocyte-derived macrophages (hMDMs) were infected with the clinically isolated M1T1 5448 strain. A decrease in the CFU count occurred between 3 and 9 h postuptake (hpu) in THP-1 cells, and no viable colonies were recovered at 24 hpu (Fig. 1A). In contrast, CFU counts decreased more slowly between 3 and 9 hpu in U937 and hMDMs and substantial bacterial numbers were recovered at 24 hpu. The CFU count reflects the net bacterial load, which is the result of bacterial replication and killing, as well as cytotoxic effects caused by bacteria. To determine if the CFU counts in these macrophages reflected killing and a replicating subpopulation of bacteria, fluorescence microscopy was used to quantify the number of intracellular bacteria in infected cells over time. M1T1 GAS grows in chains of up to 10 cocci *in vitro.* To reduce the phagocytosis of large chains (which might interfere with the quantification of intracellular bacterial replication), a low-speed centrifugation step was included prior to infection to remove longer chains of bacteria in the inoculum. Numbers of bacteria per infected cell over time were grouped into the following categories: 1 or 2, 3 to 5, 6 to 10, and ≥11. The majority (~80%) of the cells contained ≤5 bacteria at 0.5 hpu (Fig. 1B). In THP-1 macrophages, there was a steady decline in the percentages of infected cells containing 3 to 5, 6 to 10, and ≥11 bacteria between 3 and 9 hpu (Fig. 1B). In contrast, the percentage of U937 cells containing ≥11 bacteria increased significantly from 4% at 0.5 hpu to 35% at 6 and 9 hpu (Fig. 1C). Similarly, the percentage of infected hMDMs containing ≥11 bacteria increased from 2% after initial uptake to approximately 39% at 9 hpu (Fig. 1D). Representative confocal microscopy images revealed the accumulation of chains of cocci only in U937 and hMDMs between 3 and 9 h (Fig. 1E). To visualize intracellular replication within living macrophages, we imaged U937 cells infected with green fluorescent protein (GFP)-M1T1 5448 for 10 h by confocal microscopy in the presence of gentamicin to kill extracellular bacteria (for a video, see Movie S1 in the supplemental material; representative still images are shown in Fig. 1F). Infected cells containing only 1 to 5 bacteria were selected, and imaging was initiated at 2 hpu. Images were then acquired at 20-min intervals until 10 hpu. In a subpopulation of U937 cells, intracellular replication became evident from 3 and 4 hpu. From 5 hpu onward, bacteria continued to replicate, forming long chains in some cells. Collectively, these results show that M1T1 GAS can replicate in human primary macrophages and U937 cells but not in THP-1 cells.

### GAS replicates in PI-negative U937 macrophages.

**FIG 2:**
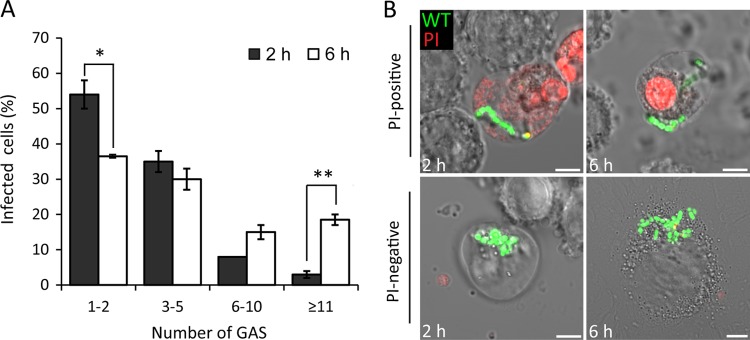
Growth of M1T1 GAS in PI-negative U937 cells. (A) Quantification of intracellular GFP-M1T1 5448 in PI-negative cells at 2 and 6 hpu. The number of bacteria in at least 50 infected cells per experiment was determined (mean ± SEM, *n* = 3). (B) Representative confocal microscopy images of PI-positive (red) or PI-negative cells infected with GFP-M1T1 5448 at the time points indicated (scale bars, 5 µm). *, *P* < 0.05; **, *P* < 0.01 (two-tailed paired t test).

Previous studies have shown that GAS induces rapid, dose-dependent apoptosis, as well as caspase-1-dependent pyroptosis, in mouse and human macrophages (16, 20). Therefore, it was possible that the observed intracellular replication was in dying cells. To measure intracellular bacterial replication in viable intact cells only, propidium iodide (PI) was included following the infection of U937 cells with M1T1 5448 GAS. PI enters cells and fluoresces only after loss of plasma membrane integrity. At 6 hpu, a significant increase in the percentage of PI-negative cells containing ≥11 bacteria was detected, compared to that seen at 2 hpu (Fig. 2A). A concomitant decrease in the percentage of PI-negative cells containing few bacteria (1-2) was observed at 6 hpu. Representative confocal microscopic images of infected PI-positive and PI-negative cells at 2 and 6 hpu are shown in Fig. 2B. Although GAS is generally regarded as being cytotoxic to macrophages, these results show that intracellular bacterial replication can occur inside macrophages with intact plasma membranes.

### SLO is required for replication of GAS in U937 macrophages.

**FIG 3:**
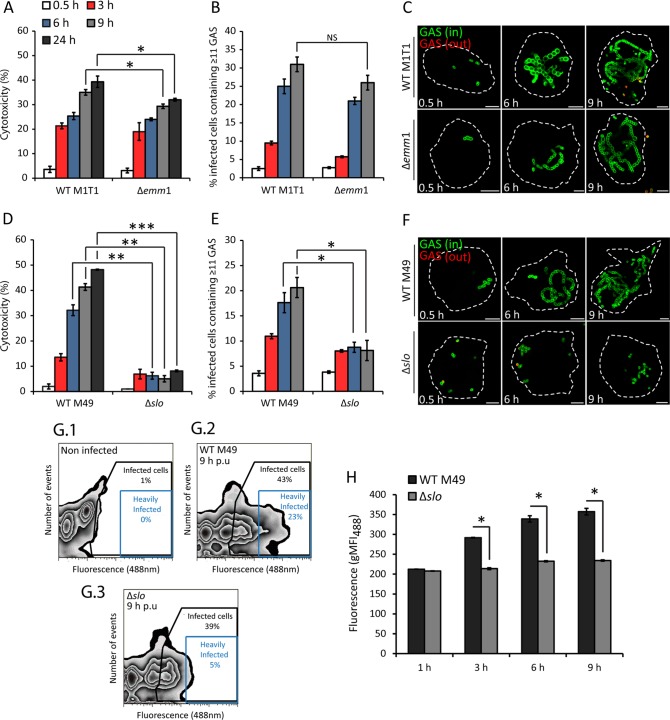
SLO is required for intracellular replication of GAS. (A) Cytotoxicity of isogenic WT M1T1 5448 and the Δ*emm1* mutant presented as a percentage of the maximum LDH release. The means ± SEM of three independent experiments in triplicate wells are shown. (B) U937 macrophages were infected with WT M1T1 5448 or the Δ*emm1* mutant, and the percentage of infected cells containing ≥11 bacteria was determined. The bacteria in at least 100 infected cells per experiment were counted. NS, not significant. (C) Representative confocal microscopy images of U937 cells infected with WT M1T1 5448 or the Δ*emm1* mutant at the time points indicated. Intracellular bacteria (in) were discriminated from extracellular bacteria (out) by differential labeling with an anti-GAS primary antibody and an Alexa Fluor 555-conjugated secondary antibody (red) without permeabilization (scale bars, 5 µm). Cell outlines are delineated by broken white lines. (D) Cytotoxicity of isogenic WT M49 NZ131 and Δ*slo* mutant strains presented as a percentage of the maximum LDH release. The means ± SEM of three independent experiments in triplicate wells are shown. (E) Cells were infected with WT M49 NZ131 or the Δ*slo* mutant, and the number of infected cells containing ≥11 bacteria was determined. The bacteria in at least 100 infected cells per experiment were counted. (F) Representative confocal microscopy images of U937 cells infected with WT M49 and the Δ*slo* mutant at the time points indicated (scale bars, 5 µm). (G.1 to G.3) Representative contour plots showing the fluorescence profile of antibody-labeled WT M49 NZ131 and the Δ*slo* mutant in U937 cells, including uninfected cells, at 9 hpu. Black boxes indicate the gating for the infected-cell population, and blue boxes indicate the gating for infected cells containing high numbers of fluorescent bacteria. (H) Geometric mean fluorescence intensity at 488 nm (gMF_488_) of WT M49 NZ131 and the Δ*slo* mutant in U937 cells across all of the time points examined. The mean ± SEM of at least three independent experiments is shown. *, *P* < 0.05; **, *P* < 0.01; ***, *P* < 0.001 (two-tailed paired t test).

The outer surface M protein is one of the best-characterized virulence factors of GAS and has been reported to promote intracellular survival in human macrophages (13). To determine if the M1 protein is required for intracellular replication under our experimental conditions, U937 cells were infected with isogenic wild-type (WT) M1T1 5448 or a Δ*emm1* mutant strain. There was a small but significant decrease in cytotoxicity at 9 and 24 hpu in the Δ*emm1* mutant compared to the WT strain (Fig. 3A). However, no difference in CFU counts (see Fig. S1 in the supplemental material) or the percentage of infected cells containing ≥11 bacteria (scored microscopically) was detected when these strains were compared (Fig. 3B and C). The intracellular growth of additional *emm*-type strains in U937 cells was also determined. The M12 and M49 NZ131 strains replicated similarly to M1T1, but the M28 and M49 CS101 strains did not (see Fig. S2A and B). This suggests that replication of GAS within U937 cells is not specific to the hypervirulent M1T1 strain.

To investigate the contribution of the pore-forming toxin SLO, a strain lacking the toxin was compared to its isogenic M49 NZ131 WT strain. As expected, the Δ*slo* mutant was considerably less toxic to U937 cells than WT M49 NZ131 was (Fig. 3D). Despite the reduced cytotoxicity, no difference between the CFU counts of the two strains was recorded (see Fig. S3 in the supplemental material). However, microscopic analysis confirmed that the Δ*slo* mutant was significantly impaired in intracellular replication compared to the isogenic WT strain. At 9 hpu, only 7% of the infected cells contained ≥11 Δ*slo* mutant bacteria while 20% contained ≥11 WT bacteria (Fig. 3E). Microscopic examination also revealed that the Δ*slo* mutant strain did not form the long replicating chains observed in the WT and Δ*emm1* strains (Fig. 3F). As an independent measure of intracellular replication, the geometric mean fluorescence intensity of antibody-labeled WT and Δ*slo* mutant GAS per infected cell was determined by flow cytometry. In agreement with the results obtained by microscopy, significantly greater replication of the WT strain than the Δ*slo* mutant was detected at 6 and 9 h, despite similar bacterial burdens at 1 hpu (Fig. 3G and H). These results show that SLO is required for replication of GAS in U937 macrophages.

### SLO reduces association of LAMP1 with GAS.

**FIG 4:**
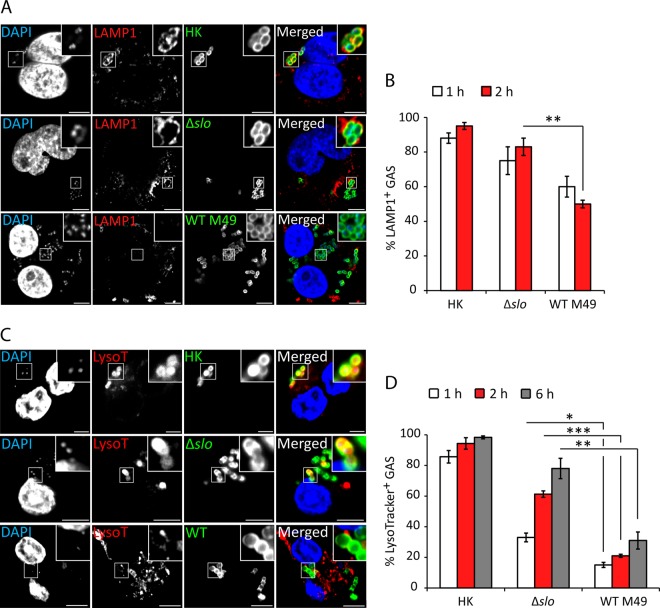
SLO reduces association of LAMP1 with GAS. (A) U937 cells were infected with live or HK WT M49 NZ131 or the Δ*slo* mutant at an MOI of 5. Bacteria were labeled with anti-GAS antibody (green) and anti-LAMP1 antibody (red), and DNA was stained with 4′,6-diamidino-2-phenylindole (DAPI; blue). Representative confocal microscopy images obtained at 2 hpu are shown with higher magnifications of boxed areas (scale bars, 5 µm). (B) Quantification of the colocalization of the GAS strains indicated and LAMP1. Data represent the results of at least 100 infected cells in each of three independent experiments (mean ± SEM). (C) U937 cells were infected with live or HK WT M49 NZ131 or the Δ*slo* mutant at an MOI of 5. LysoTracker dye was added to infected cells 15 min prior to each time point. Bacteria were labeled with anti-GAS antibody (green). LysoTracker-positive acidic compartments are red, and DNA stained with DAPI is blue. Representative confocal microscopy images obtained at 2 hpu are shown with higher magnifications of boxed areas (scale bars, 5 µm). (D) Quantification of GAS colocalization with LysoTracker dye. Data shown represent the results of at least 100 infected cells in three independent experiments (mean ± SEM). *, *P* < 0.05; **, *P* < 0.01; ***, *P* < 0.001 (two-tailed paired t test).

Fusion of lysosomes to the phagosome is an essential microbicidal mechanism of macrophages (21). Reports on the intracellular localization and trafficking of GAS vary widely, possibly reflecting strain variations and different analytical techniques. The localization of WT M49 NZ131 and Δ*slo* mutant strains after uptake into U937 macrophages was determined by labeling infected cells for LAMP1. Over 95% of heat-killed (HK) bacteria were at least partially surrounded by LAMP1 at 2 hpu. In Δ*slo* mutant-infected cells, approximately 80% of the GAS bacteria were associated with LAMP1, whereas in cells infected with WT bacteria, the association was significantly reduced, to 50% at 2 hpu (Fig. 4A and B). An acidotropic LysoTracker dye was then used to label phagolysosomes. Whereas the majority of WT bacteria did not colocalize with LysoTracker, approximately 60% of the Δ*slo* mutant-containing GCVs had undergone acidification at 2 hpu (Fig. 4C and D). The finding that approximately 50% of the WT GAS bacteria were LAMP1 associated at 2 hpu but only 21% were colabeled with LysoTracker suggests that GAS might prevent phagolysosomal maturation, as well as mediate escape from the GCV.

### SLO is required for GCV rupture and bacterial escape into the cytosol.

**FIG 5:**
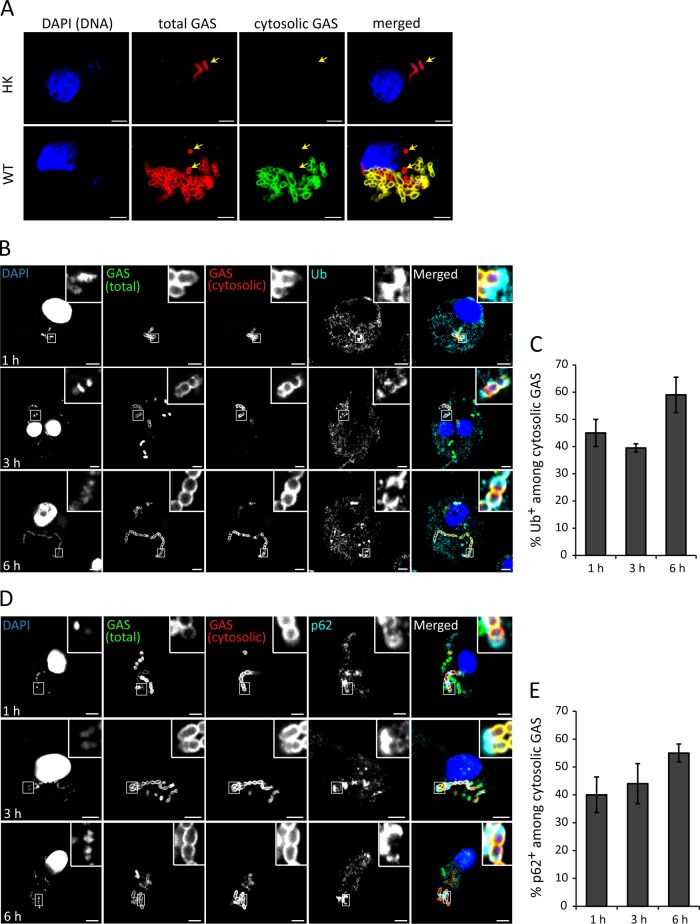
GAS bacteria rupture the GCV and escape into the macrophage cytosol. (A) Representative confocal microscopy images of U937 cells infected with HK or WT M49 NZ131 GAS at an MOI of 0.5. Cytosolic bacteria were labeled following digitonin-mediated semipermeabilization of the plasma membrane (green), followed by labeling of total bacteria (red) with saponin at 6 hpu. Yellow arrows indicate vacuolar bacteria. DNA was stained with DAPI (blue) (scale bars, 5 µm). (B) Representative confocal microscopy images of cytosolic M49 NZ131 GAS immunolabeled with antiubiquitin antibodies in digitonin-treated U937 cells at the time points indicated. Ub, ubiquitin. (C) Quantification of ubiquitin colocalization among cytosolic GAS bacteria. At least 100 infected cells were scored in at least three independent experiments (mean ± SEM). (D) Representative confocal microscopy images of cytosolic M49 NZ131 GAS immunolabeled with anti-p62 antibodies in digitonin-treated U937 cells. (E) Quantification of p62 colocalization among cytosolic bacteria. At least 100 infected cells were scored in at least three independent experiments (mean ± SEM).

The reduced association of LAMP1 with WT GAS but not the Δ*slo* mutant might also be due to SLO-mediated bacterial escape from the phagosome. Therefore, we tested if cytosolic GAS was present in U937 macrophages. Digitonin was used at a concentration that selectively permeabilized the plasma membrane so that only cytosolic GAS bacteria were immunolabeled with an anti-GAS antibody. The luminal contents of intact phagosomes remained inaccessible to the antibody, as indicated by the yellow arrows in Fig. 5A. HK bacteria were very rarely observed in the cytosol (Fig. 5A, top). In contrast, WT M49 NZ131 bacteria were more readily found in the cytosol (Fig. 5A, bottom), confirming the presence of cytosolic GAS within U937 macrophages. Ubiquitin frequently decorates cytosolic bacteria (22, 23), acting as a signal for autophagy receptors such as p62 to direct the antibacterial autophagy machinery toward such bacteria. Indeed, antibacterial autophagy has been reported to restrict the growth of GAS in epithelial cells (18, 24). To determine the proportion of cytosolic GAS that was recognized in the cytosol by the ubiquitin-mediated host detection system, U937 cells were infected with GAS strain M49 NZ131 for different time periods, exposed to digitonin, and then immunolabeled for ubiquitin or p62 prior to fixation (Fig. 5B to E). Approximately 45% of the cytosolic bacteria colocalized with ubiquitin at 1 hpu (Fig. 5C). A similar percentage (40%) of ubiquitin colocalization was found at 3 hpu, and by 6 hpu, more than half (59%) of the cytosolic bacteria were associated with ubiquitin. Approximately 40% of the cytosolic GAS bacteria colocalized with the ubiquitin-LC3 cargo receptor p62 at 1 hpu, similar to levels of ubiquitin colocalization (Fig. 5E). These data confirm the presence of cytosolic WT GAS within U937 cells and suggest that approximately 50% of such bacteria might be targeted for antibacterial autophagy.

**FIG 6:**
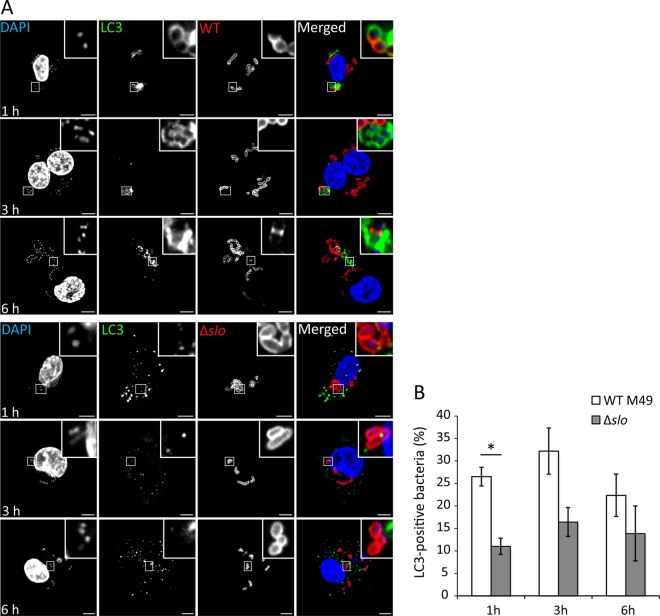
SLO stimulates targeted autophagy. (A) Representative confocal microscopy images of GFP-LC3 (green) U937 cells infected with WT M49 NZ131 GAS and the Δ*slo* mutant. Bacteria were labeled with anti-GAS antibody (red) and DNA was stained with DAPI (blue) at the time points indicated. (B) Quantification of LC3 colocalization with WT and Δ*slo* mutant bacteria. At least 100 infected cells were scored in at least three independent experiments (mean ± SEM). The region of colocalization is magnified (boxed areas) (scale bars, 5 µm). *, *P* < 0.05; two-tailed paired *t* test.

As various strains of GAS are efficiently targeted for degradation in LC3-positive autophagosomes within epithelial cells (8, 25), the proportion of GAS bacteria within autophagosomes was quantified in U937 macrophages. GFP-LC3 colocalization with total bacteria was quantified after permeabilization with saponin, since bacteria enclosed in an autophagosome would no longer be cytosolic. We found that 26% of the total intracellular bacteria were associated with LC3 at 1 hpu and that LC3 recruitment was significantly lower in Δ*slo* mutant-infected cells, consistent with SLO-mediated rupture of the phagosome (Fig. 6A and B). Slightly lower levels of LC3-positive GAS bacteria of the M1T1 5448 strain were also observed, suggesting that this finding is not strain type specific (see Fig. S4 in the supplemental material).

### GAS replicates in the cytosol of U937 macrophages.

**FIG 7:**
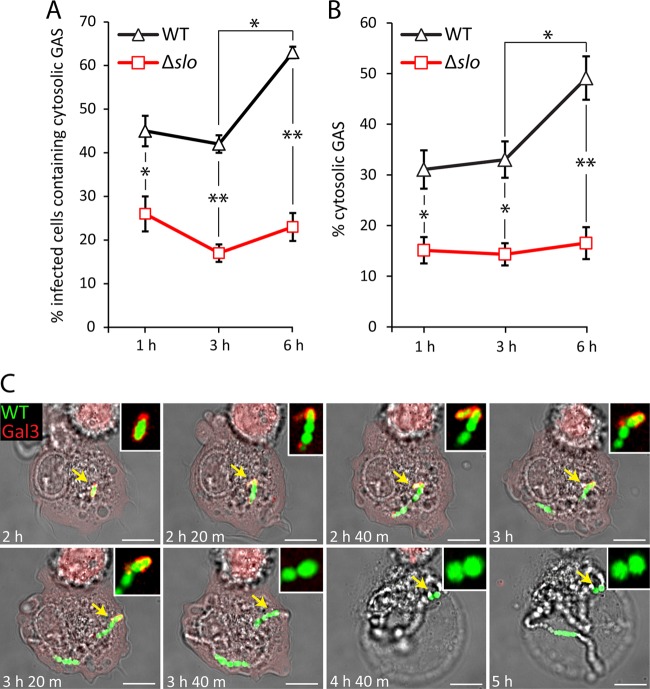
Replication of GAS after rupture of the GCV. U937 cells were infected with M49 NZ131 or Δ*slo* GAS at an MOI of 0.5 and permeabilized with digitonin to quantify the infected cells containing at least one cytosolic bacterium (A) or the total cytosolic bacteria in infected cells (B) at 1, 3, and 6 hpu. The mean ± SEM of at least three independent experiments is shown (scale bars, 5 µm). *, *P* < 0.05; **, *P* < 0.01 (two-tailed paired t test). (C) Stills from live confocal imaging of mCherry-tagged galectin 3-expressing U937 cells infected with GFP-M1T1 5448 GAS at an MOI of 0.5. Imaging was initiated at 2 hpu, and the cells were imaged every 20 min until 10 hpu. The elapsed time is shown at the bottom left of each image. Each yellow arrow highlights a single coccus that replicates to form a long chain, as well as its association with mCherry galectin 3. This region is magnified and shown in the white box in the upper right of each image. The still images are representative of 22 infected cells in four independent live-imaging experiments (scale bars, 5 µm).

One recent study reported that M1T1 GAS avoids recognition of autophagy in epithelial cells (26). Since not all cytosolic bacteria were targeted for ubiquitin- and p62-mediated antibacterial autophagy, this suggests that a subpopulation of cytosolic GAS within macrophages is not recognized for autophagy-mediated growth attenuation. Furthermore, we noted that cytosolic WT GAS bacteria were often found in large numbers within one cell (Fig. 5A), which could indicate cytosolic bacterial replication. To test this more directly, the percentages of infected U937 cells containing at least one cytosolic bacterium were quantified at 1, 3, and 6 hpu (Fig. 7A). As our results with LAMP1 labeling suggested that SLO mediates escape from the phagosome, the proportion of cytosolic Δ*slo* mutant bacteria was also quantified. Cytosolic WT bacteria were present in 45% of the infected cells at 1 hpu and in 64% of them at 6 hpu. This was significantly higher than the percentage of cells containing cytosolic Δ*slo* mutant bacteria, which ranged from 20 to 25% between 1 and 6 h. Next, the percentages of cytosolic WT and Δ*slo* mutant bacteria were quantified (Fig. 7B). Cytosolic bacteria accounted for 31 and 33% of the total WT bacteria at 1 and 3 hpu, respectively. At 6 hpu, the earliest time point of replication, the percentage of cytosolic bacteria increased significantly to 49%. In contrast, the percentage of cytosolic Δ*slo* mutant bacteria ranged from 14 to 17% between 1 and 6 hpu, significantly less than cytosolic WT bacteria at each time point. Finally, when infected cells containing ≥20 WT bacteria were analyzed, 84.6% ± 3.2% (mean ± standard error of the mean [SEM] of three experiments) of the bacteria were cytosolic. Together, this indicates that GAS undergoes replication in the cytosol following vacuole rupture.

Time-lapse imaging of GFP-M1T1 5448-infected U937 cells expressing mCherry-tagged galectin 3 (27) was used to investigate more directly the fate of GAS after damage of the vacuole and entry into the macrophage cytosol (for a representative video, see Movie S2 in the supplemental material; still images are shown in in Fig. 7C). Infected cells containing only 1 or 2 galectin 3-associated bacteria were selected, and imaging was initiated at 2 hpu. Images were acquired at 20-min intervals until 10 hpu. In 31% of the selected cells, the formation of a rapidly expanding coccal chain was observed after the recruitment of galectin 3 to the damaged vacuole. From 2 to 4 hpu, a single coccus replicated to form a long chain consisting of >20 cocci. As the bacteria replicated, a residual galectin 3 signal was apparent at the poles of the expanding coccal chain (indicated by arrows); this was most likely to be remnants of the ruptured vacuolar membrane. Bacterial replication was frequently followed by loss of normal macrophage morphology and galectin 3 fluorescence, consistent with a loss of membrane integrity and resulting cell death.

Collectively, these results indicate that SLO-mediated rupture of the GCV membrane generates a significant proportion of cytosolic bacteria. Some of the cytosolic bacteria are then recognized by ubiquitin and the autophagy receptor p62, resulting in LC3 association with bacteria. However, antibacterial autophagy appears to be insufficient to fully control bacterial replication, as shown by the presence of cells containing large numbers of cytosolic bacteria and live imaging of GAS replicating in the host cell cytosol.

## DISCUSSION

Although GAS is a major human-adapted bacterial pathogen, surprisingly little is known about its fate in human macrophages. To date, only one study has provided tentative evidence that GAS can survive and replicate in human macrophages (13). In many GAS-host cell interaction studies (13, 16, 19), CFU assays have been used to measure the survival of the total GAS population, which includes the intracellular bacteria, as well as the extracellularly released bacteria recovered during a period after antibiotic withdrawal from the cell culture medium. This technique therefore fails to distinguish between intracellular replication and extracellular replication after host cell lysis. Under our conditions, where extracellular bacterial replication was inhibited by the continued presence of antibiotic, GAS bacteria were found to survive longer in U937 and primary macrophages than in THP-1 macrophages. However, CFU counts reflect both replication and killing sustained by the bacteria and host cell cytotoxic effects, which could expose intracellular bacteria to antibiotic in the medium, resulting in their death. For these reasons, CFU counts do not necessarily represent an accurate measurement of intracellular survival and/or replication of GAS. Therefore, we used fluorescence microscopy to count bacteria and showed that GAS can replicate within U937 and primary macrophages but not in THP-1 cells. We identified a subpopulation of macrophages with hyperreplicating GAS (defined as ≥11 GAS bacteria per cell), including some cells that were packed with numerous bacteria. The extensive replication and striking formation of long GAS chains were also observed by time-lapse microscopy and flow cytometry in U937 macrophages. Since GAS induces macrophage cytotoxicity (16), we quantified growth from 2 to 6 hpu in PI-negative cells and showed that GAS can replicate in macrophages before the onset of host cell death. Moreover, during microscopic quantification of infected cells, intracellular bacteria were differentiated from extracellular bacteria by labeling with anti-GAS antibody without permeabilization of the host cell membrane. Since only cells with intact cell membranes can restrict the entry of extracellular antibodies, this provides indirect evidence that the majority of the cells containing intracellular replicating bacteria (≥11) were viable.

The ability of GAS to replicate efficiently in U937 cells was not specific to the hypervirulent M1T1 serotype but was also found in an M12 throat isolate and the M49 NZ131 nephritogenic strain (28). This suggested that the presence of the M1 protein, previously reported to mediate resistance to phagocytic killing *in vitro* (13, 29), was not required to enable bacterial replication in our experiments. Furthermore, we found no difference in survival or replication between the WT and the Δ*emm1* mutant in U937 macrophages. Instead, we found an essential role for SLO in promoting the intracellular replication of NZ131 M49 GAS. Although the Δ*slo* mutant was defective for intracellular replication and caused significantly less cytotoxicity than the WT M49 strain, no significant difference in CFU counts were recorded. This further illustrates the limitations of population-based methods in examining the contribution of GAS virulence factors to intracellular survival and/or replication. Despite exerting a cytotoxic effect, several studies have reported that expression of SLO increases the recovery of GAS from epithelial cells compared to that of a SLO-deficient strain (16, 30). However, the mechanism by which SLO promotes survival is uncertain. It has been reported that GAS lacking SLO undergoes enhanced interactions with late endosomal acidified compartments compared to the WT strain, leading to the suggestion that SLO prevents lysosomal fusion to the GCV (4, 17). However, this might also be explained by a larger cytosolic population of WT GAS bacteria than of a SLO-deficient strain. Indeed, GAS bacteria are exposed to the cytosol in skin keratinocytes and SLO pores can promote the survival of GAS in autophagosome-like structures, which eventually fuse to become autolysosomes but at a lower rate than that of a SLO mutant (30). Finally, as two independent studies failed to observe cytosolic GAS in human macrophages, it remains possible that, under certain conditions, GAS could replicate in a modified vacuole throughout infection (13, 19). We found that approximately 60% of the WT bacteria were associated with LAMP1 at 1 hpu in U937 macrophages while <20% of the WT GCVs underwent acidification at this time point. This suggests that GAS might prevent acidification of the GCV (19), as well as promote bacterial entry into the nutrient-rich cytosol of U937 macrophages, through SLO. In support of SLO-dependent GCV rupture, digitonin-mediated permeabilization of the plasma membrane revealed that at 6 hpu, 49% of the WT GAS bacteria were cytosolic, compared to 17% of the Δ*slo* mutant GAS bacteria. In the complete absence of SLO, cytotoxicity, vacuole rupture, and bacterial replication were drastically reduced. Nevertheless, a proportion of the SLO-expressing GAS bacteria failed to rupture the GCV and replicate. This might be due to variations in the level of SLO produced by different intracellular bacteria.

Several previous studies have shown that selective autophagy can restrict the growth of M6 (18), M49 (31), M89 (24), and M3 (30) strains of GAS. However, it was reported recently that the M1T1 5448 strain avoids recognition by autophagy proteins such as ubiquitin and NDP52, preventing LC3-mediated autophagosome recruitment (26). This was attributed to the proteolytic activity of the cysteine protease SpeB. As a result of impaired autophagic recognition, this strain was found to replicate in the cytosol of HeLa cells. Since the strains used in our study express SpeB, we tested whether autophagy proteins were recruited to cytosolic bacteria in U937 macrophages. We found that between 1 and 6 hpu, 40 to 50% of the cytosolic M49 NZ131 bacteria were recognized by ubiquitin and p62, and at 3 hpu, 32 and 24% of the total WT M49 and M1T1 GAS bacteria associated with LC3, respectively. These results are different from the findings of Barnett et al., who showed that at 4 hpu in HeLa cells, only 3% of the WT M1T1 GAS bacteria associated with LC3, whereas 26% of the Δ*speB* mutant bacteria were associated with this autophagy marker (26). Therefore, while SpeB-mediated avoidance of autophagy is active in epithelial cells, it appears to have a minor effect in macrophages. Whether other virulence factors support the growth of cytosolic GAS in macrophages by disrupting autophagic signaling or downstream maturation/fusion events needs to be investigated further.

A recent study showed that GAS replicates in nonacidic LAMP1- and LC3-positive vacuoles in endothelial cells (32). In contrast, we found that where cells contained a large number of bacteria, they were predominantly cytosolic. Furthermore, time-lapse microscopy of infected galectin 3-expressing U937 cells revealed that rupture of the GCV was followed immediately by bacterial replication in the cytosol. Strikingly, the galectin signal was frequently localized at both ends of the expanding coccal chain, suggesting that remnants of the ruptured membrane are forced away from dividing bacteria. This shows that replication of GAS in human macrophages occurs predominantly within the cytosol.

## MATERIALS AND METHODS

### Bacterial strains and growth conditions.

GAS strain 5448 is a clinical isolate obtained from a patient with NF and STSS and is representative of the globally disseminated M1T1 clone (33). WT M1T1 5448, GFP-5448, and the mutant lacking the M1 protein (Δ*emm1*) were provided by A. Norrby-Teglund (13). The serotype M49 NZ131 strain is a skin isolate from a patient with glomerulonephritis (28). S. Sriskandan provided NZ131, the mutant lacking the SLO toxin (Δ*slo*), an M28 clinical throat isolate, and the M49 CS101 strain first isolated and characterized by P. Cleary. GAS strains were routinely grown at 37°C in a standing culture for 16 h until stationary phase in Todd-Hewitt broth (THB; Oxoid) supplemented with 1% yeast extract.

### Antibodies and reagents.

Macrophage colony-stimulating factor (MCSF; ImmunoTools), phorbol 12-myristate 13-acetate (PMA; Sigma), LysoTracker Red DND-99 (Molecular Probes), PI (Life Technologies), heat-inactivated human serum (Sigma), and Accutase (Sigma) were used. The primary antibodies were a goat anti-group A polysaccharide antibody (Abcam), a mouse anti-LAMP1 antibody (BD Bioscience), a mouse anti-ubiquitin antibody (FK2; Enzo), and a mouse anti-SQSTM1 (p62) antibody (Abcam). The secondary antibody was an Alexa Fluor-conjugated donkey anti-mouse or donkey anti-goat antibody (Invitrogen).

### Cell culture and primary monocyte isolation.

The THP-1 and U937 human monocytic cell lines were originally obtained from the ATCC. THP-1 cells were maintained at 2 × 10^5^/ml in RPMI medium (Gibco) supplemented with 10% heat-inactivated fetal calf serum (FCS; Invitrogen), 2 mM l-glutamine (Sigma), 10 mM HEPES (Sigma), 1 mM sodium pyruvate (Sigma), 2.5 g/liter glucose (Sigma), and 0.05 mM β-mercaptoethanol (Sigma) at 37°C in 5% CO_2_. U937 cells were maintained at 1 × 10^6^/ml of RPMI medium supplemented with 10% FCS and 2 mM l-glutamine. To obtain a macrophage-like state, THP-1 and U937 cells were differentiated in 25 ng/ml PMA for 3 days. Peripheral blood mononuclear cells (PBMCs) were isolated from the blood of healthy human volunteers after informed consent and in accordance with the ethical guidelines of Imperial College, London. PBMCs were isolated by density gradient centrifugation with Ficoll-Paque Plus (GE Healthcare) and transferred to RPMI medium containing 10% FCS supplemented with 100 U/ml penicillin-streptomycin (Sigma). Red blood cells (RBC) were lysed in Hybri-Max RBC-lysing buffer (Sigma) for 10 min at 37°C, centrifuged, and resuspended in RPMI medium. Cells were counted and subjected to CD14-positive selection (Miltenyi Biotec), according to the manufacturer’s instructions. CD14-positive monocytes were differentiated in 20 ng/ml MCSF and seeded onto 24-well plates at 2 × 10^5^/ml. MCSF was removed after 3 days and replaced with fresh RPMI medium for a further 4 days. Cells were cultured in antibiotic-free medium 24 h prior to infection.

### Macrophage infection.

Differentiated macrophages were cultured in 24-well plates at a density of 2 × 10^5^/well or in 6-well plates at 1 × 10^6^/well. GAS was grown until stationary phase at 37°C in THB. Prior to infection, long chains of GAS bacteria were removed from the infection inoculum by a precentrifugation step as follows. The bacterial culture was pelleted and resuspended in warm RPMI containing 10% FCS. One milliliter of culture was added to each Eppendorf tube and pelleted at 1,000 × *g* for 1 min. The top half of each supernatant (containing an enriched mixture of single-coccus, diplococcus, and small-chain-forming GAS) was combined and pelleted at top speed. Each pellet was resuspended in RPMI and concentrated into a single tube. Bacteria were opsonized in 10% heat-inactivated human serum at room temperature for 20 min. Bacteria were added to the cells at a multiplicity of infection (MOI) of 5 or 0.5 and centrifuged at 110 × *g* for 5 min to increase and synchronize infection. After incubation for 30 min at 37°C in 5% CO_2_, cells were washed twice and incubated with 100 µg/ml gentamicin for 1 h and then with 25 µg/ml gentamicin thereafter.

### Bacterial survival and cytotoxicity.

Bacterial intracellular survival was determined by CFU counting on THB agar plates. At selected times postuptake, cells were washed three times with PBS and lysed in 0.05% Triton X-100 in phosphate-buffered saline (PBS) for 5 min. The cell lysates was serially diluted in PBS, and the number of CFU was determined after plating onto THB agar plates. Bacterial survival was measured as the total number of CFU per milliliter. Cytotoxicity was determined by the lactate dehydrogenase (LDH) release assay. Prior to infection, the normal cell culture medium was replaced with phenol-free RPMI medium (Gibco) containing the appropriate supplements, as previously described. Cells were infected at an MOI of 0.5, and at selected times postuptake, cell culture supernatants were removed from each well and centrifuged to pellet any suspended cells. The supernatant was removed and kept at −20°C until all samples were collected. Cytotoxicity was determined by measuring the amount of LDH released into the cell culture medium with the CytoTox 96 nonradioactive cytotoxicity assay (Promega), according to the manufacturer’s instructions. Fifty microliters of supernatant was mixed with 50 µl of substrate reagent and incubated at room temperature in the dark. Absorbance at 490 nm was measured, and cytotoxicity was calculated as follows: (sample LDH release − spontaneous LDH release)/(maximum LDH release − spontaneous LDH release) × 100. The maximum LDH release was determined by freezing replicate uninfected wells at −80°C for 30 min and harvesting the supernatants after thawing. The spontaneous LDH release was determined by harvesting supernatants from uninfected wells throughout the experiment.

### Immunofluorescence microscopy.

For visualization and quantification of intracellular bacteria, differentiated macrophages were seeded onto glass coverslips in 24-well plates and infected with GAS strains as described previously. At the selected times postuptake, coverslips were washed and fixed in 3% paraformaldehyde (PFA) in PBS for 15 min. Primary antibodies were diluted to the appropriate working concentration in 10% horse serum (HS) in PBS containing the permeabilizing agent saponin (0.1%) and added to the cells for 1 h. Cells were washed twice in PBS and incubated with an Alexa Fluor-conjugated secondary antibody containing 10% HS with 0.1% saponin. Cells were washed twice in PBS and once in distilled H_2_O and mounted onto a microscope slide. Fixed cells were imaged with a laser-scanning microscope (LSM710; Zeiss). Acquired images were processed and false colored with the Zeiss imaging software or Adobe Illustrator software. For bacterial in/out labeling, extracellular bacteria were labeled with primary (anti-GAS) and secondary antibodies without permeabilization, and then intracellular bacteria were differentially labeled with an anti-GAS primary antibody and a different Alexa Fluor-conjugated secondary antibody in the presence of saponin. In GFP-M1T1 5448-infected cells, extracellular bacteria were labeled with an anti-GAS antibody and a Alexa Fluor 555-conjugated secondary antibody in the absence of permeabilization. For LysoTracker Red labeling, infected cells were incubated with 40 nM LysoTracker probe for 15 min prior to fixation.

### Flow cytometry.

U937 cells were seeded in six-well plates at 1 × 10^6^/ml, differentiated with PMA for 3 days, and infected with the isogenic M49 NZ131 or Δ*slo* mutant GAS strain as described previously. At the selected time postuptake, cells were washed and detached with Accutase for 15 min and centrifuged at 250 × *g* for 5 min. Cells were resuspended in 3% PFA in PBS for 15 min and then centrifuged. The fixed cells were resuspended in 10% HS in PBS-saponin containing anti-GAS primary antibody for 30 min, and then cells were washed twice by centrifugation and resuspension in PBS and incubated in the secondary antibody for 30 min. After labeling, cells were resuspended in PBS for analysis by flow cytometry. Analysis was carried out with a two-laser, four-color FACSCalibur flow cytometer (BD Biosciences). Uninfected cells were used to calibrate the instrument settings. Fluorescence-activated cell sorting data were analyzed with FlowJo software version 7.6.5 (TreeStar).

### Live imaging.

WT U937 cells or U937 cells stably expressing mCherry-tagged galectin 3 were differentiated on a 35-mm glass-bottom dish 3 days prior to infection with GFP-M1T1 5448 GAS as described previously. At 1 h 30 min prior to imaging, the medium was replaced with Opti-MEM (Gibco) imaging medium supplemented with 5% FCS, 10 mM HEPES, and 25 µg/ml gentamicin. The dish was transferred to the confocal microscope and mounted onto an automated stage fully enclosed within a 37°C live-cell chamber. The dish was left undisturbed for 20 min to maintain temperature stability within the chamber. Live imaging with a 63× objective was initiated at 2 hpu and continued until 10 hpu. Multipositional acquisition was used to select and record approximately 20 to 30 different fields of view that contained infected cells with at least one bacterium or one galectin-positive GCV. Images were acquired every 20 min for each selected position by means of the automated motorized stage. Imaging movies were processed with ImageJ and Fiji software.

### Digitonin permeabilization.

To label and quantify intracellular cytosolic bacteria, differentiated U937 macrophages were infected with the isogenic M49 NZ131 or Δ*slo* mutant GAS strain as described previously. At the selected time postuptake, cells were washed twice in PBS and incubated on ice for 2 to 3 min. Cells were gently washed twice in ice-cold sterile KHM buffer (110 mM potassium acetate, 20 mM HEPES, 2 mM MgCl_2_ in PBS) and incubated in 500 µl of ice-cold digitonin in KHM buffer at concentrations of 20, 40, and 50 µg/ml for approximately 5 min on ice. Cells were gently washed once with ice-cold KHM buffer and once more with ice-cold PBS. To label the cytosolic bacteria, the primary anti-GAS antibody was prepared in 10% HS in PBS and 200 µl was transferred onto the cells for 30 min at room temperature. Cells were gently washed twice in PBS and fixed with 3% PFA for 10 min. Cells were washed twice in PBS and incubated in secondary antibody in 10% HS--PBS for 1 h. To label the total bacteria (vacuolated and cytosolic), the cells were washed and incubated first with an anti-GAS primary antibody and then with a different Alexa Fluor-conjugated secondary antibody in 10% HS--PBS with 0.1% saponin. Each experiment was carried out with three different concentrations to assess the efficacy of selective permeabilization, and HK bacteria were used as the internal negative control. Each experiment was carried out in duplicate with three independent repeats.

### Statistical analysis.

Data are expressed as mean ± SEM. Statistical testing was carried out with two-tailed Student *t* tests of data from experimental assays repeated at least three times. *P* values of <0.05 were considered statistically significant.

## SUPPLEMENTAL MATERIAL

Movie S1  Time-lapse microscopy video of U937 cells infected with GFP-M1T1 5448 at an MOI of 0.5. Live imaging was initiated at 2 hpu, and images were acquired at 20-min intervals until 10 hpu. Download Movie S1, AVI file, 2.6 MB

Movie S2  Time-lapse microscopy video of mCherry-tagged, galectin 3-expressing U937 cells infected with GFP-M1T1 5448 at an MOI of 0.5. Live imaging was initiated at 2 hpu, and images were acquired at 20-min intervals until 10 hpu. Download Movie S2, AVI file, 17.5 MB

Figure S1  Survival of the WT and Δ*emm1* mutant strains. U937 cells were infected with isogenic WT M1T1 5448 and the Δ*emm1* mutant at an MOI of 5, and intracellular bacteria were measured by CFU counting at the time points indicated. The mean ± SEM of three independent experiments in triplicate wells and duplicate colony counts is shown. Download Figure S1, TIF file, 1.7 MB

Figure S2  Cytotoxicity and replication of different *emm*-type strains in U937 cells. (A) Cytotoxicity of M12, M28, M49 CS101, and M49 NZ131 strains presented as a percentage of the maximum LDH release. The mean ± SEM of at least three independent experiments in triplicate wells is shown. (B) The percentage of infected cells containing ≥11 bacteria was scored. The bacteria in at least 50 infected cells per experiment were counted (mean ± SEM, *n* = 3). Download Figure S2, TIF file, 2.8 MB

Figure S3  Survival of WT and Δ*slo* deletion mutant strains. U937 cells were infected with isogenic WT M49 NZ131 and the Δ*slo* mutant at an MOI of 5, and intracellular bacteria were measured by CFU counting at the time points indicated. The mean ± SEM of three independent experiments in triplicate wells and duplicate colony counts is shown. Download Figure S3, TIF file, 2.1 MB

Figure S4  M1T1 5448 GAS colocalizes with GFP-LC3 in U937 cells. (A) Representative confocal microscopy images of GFP-LC3 (green) U937 cells infected with WT M1T1 5448 GAS. Bacteria were labeled with anti-GAS antibody (red) and DNA was stained with DAPI (blue) at the time points indicated (scale bars, 5 µm). (B) Quantification of LC3 colocalization to WT M1T1 5448. At least 100 infected cells were scored in at least three independent experiments (mean ± SEM). Regions of colocalization are magnified and shown in boxed areas. Download Figure S4, TIF file, 2.6 MB
