# Erratum for O’Neill et al., Cytosolic Replication of Group A *Streptococcus* in Human Macrophages

**DOI:** 10.1128/mBio.00931-16

**Published:** 2016-06-28

**Authors:** Alan M. O’Neill, Teresa L. M. Thurston, David W. Holden

**Affiliations:** MRC Centre for Molecular Bacteriology and Infection, Imperial College London, United Kingdom

## ERRATUM

Volume 7, no. 2, doi:10.1128/mBio.00020-16, 2016. We inadvertently omitted to acknowledge the generosity of Joseph Ferretti for providing the M49 NZ131 strain and a mutant derivative lacking the SLO toxin (**D. J. Savic, W. M. McShan, and J. J. Ferretti.** 2002. Autonomous expression of the *slo* gene of the bicistronic *nga*-*slo* operon of *Streptococcus pyogenes*. Infect Immun **70:**2730–2733. http://dx.doi.org/10.1128/IAI.70.5.2730-2733.2002).

