# Supplementary figures and images for: Cytosolic Replication of Group A Streptococcus in Human Macrophages
Source: mBio. 2016 Apr 12;7(2):e00020-16. doi: 10.1128/mBio.00020-16 (PMC4959517; doi:10.1128/mBio.00020-16)

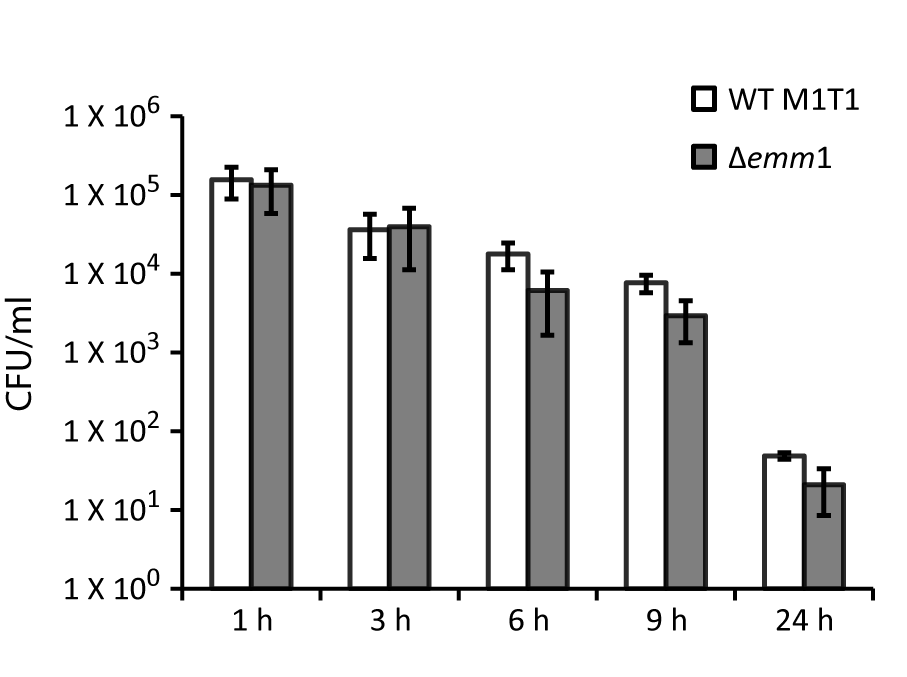

Supplement: Figure S1 — Survival of the WT and Δemm1 mutant strains. U937 cells were infected with isogenic WT M1T1 5448 and the Δemm1 mutant at an MOI of 5, and intracellular bacteria were measured by CFU counting at the time points indicated. The mean ± SEM of three independent experiments in triplicate wells and duplicate colony counts is shown. Download [file mbo002162766sf1.tif]

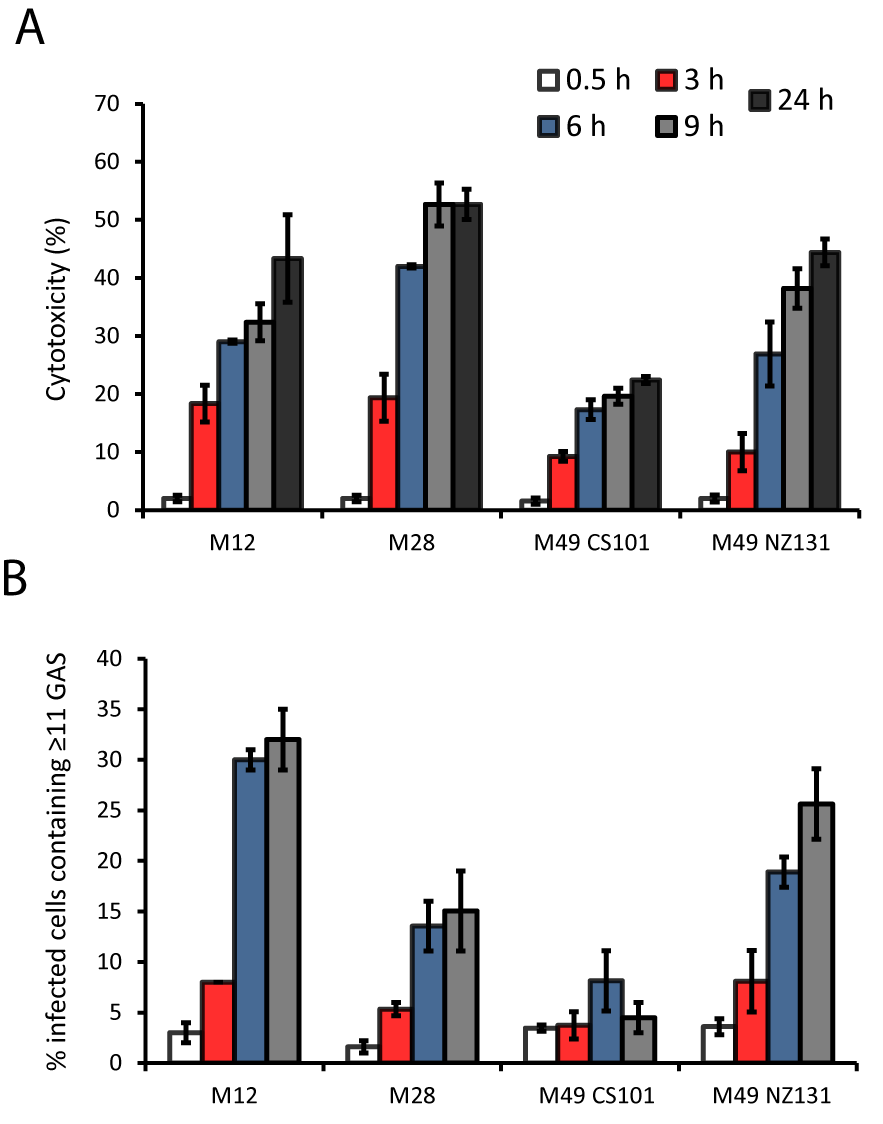

Supplement: Figure S2 — Cytotoxicity and replication of different emm-type strains in U937 cells. (A) Cytotoxicity of M12, M28, M49 CS101, and M49 NZ131 strains presented as a percentage of the maximum LDH release. The mean ± SEM of at least three independent experiments in triplicate wells is shown. (B) The percentage of infected cells containing ≥11 bacteria was scored. The bacteria in at least 50 infected cells per experiment were counted (mean ± SEM, n = 3). Download [file mbo002162766sf2.tif]

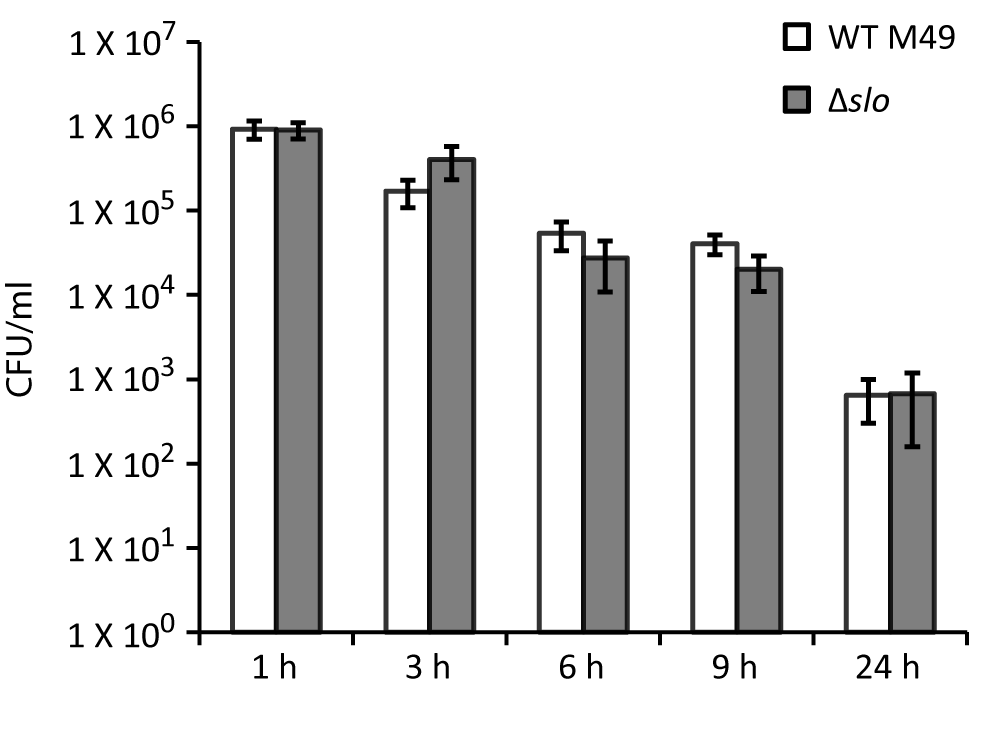

Supplement: Figure S3 — Survival of WT and Δslo deletion mutant strains. U937 cells were infected with isogenic WT M49 NZ131 and the Δslo mutant at an MOI of 5, and intracellular bacteria were measured by CFU counting at the time points indicated. The mean ± SEM of three independent experiments in triplicate wells and duplicate colony counts is shown. Download [file mbo002162766sf3.tif]

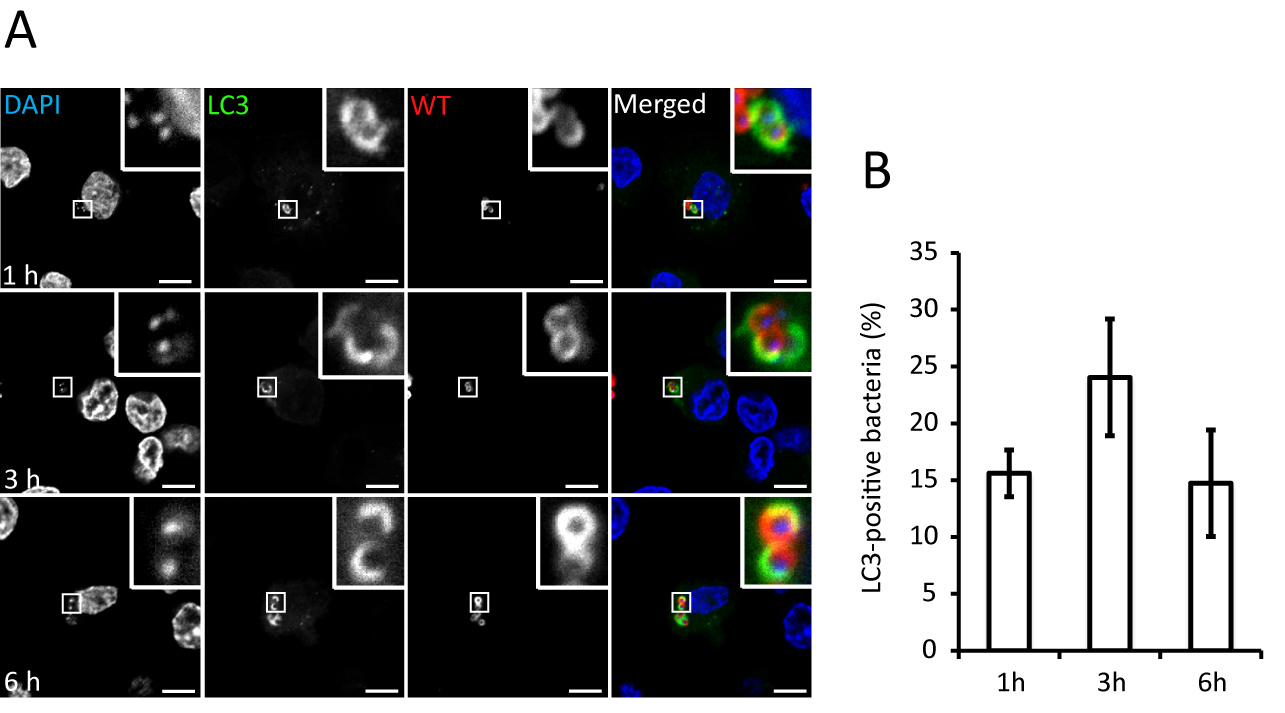

Supplement: Figure S4 — M1T1 5448 GAS colocalizes with GFP-LC3 in U937 cells. (A) Representative confocal microscopy images of GFP-LC3 (green) U937 cells infected with WT M1T1 5448 GAS. Bacteria were labeled with anti-GAS antibody (red) and DNA was stained with DAPI (blue) at the time points indicated (scale bars, 5 µm). (B) Quantification of LC3 colocalization to WT M1T1 5448. At least 100 infected cells were scored in at least three independent experiments (mean ± SEM). Regions of colocalization are magnified and shown in boxed areas. Download [file mbo002162766sf4.tif]
